# Reprogrammed Plant Metabolism During Viral Infections: Mechanisms, Pathways and Implications

**DOI:** 10.1111/mpp.70066

**Published:** 2025-02-19

**Authors:** Tong Jiang, Tianwen Hao, Wenjing Chen, Chengliang Li, Shuqi Pang, Chenglong Fu, Jie Cheng, Chaobo Zhang, Mansour Ghorbanpour, Shuo Miao

**Affiliations:** ^1^ College of Agriculture and Biology Liaocheng University Liaocheng China; ^2^ Shandong Meng'en Modern Agriculture Development Co. Ltd. Liaocheng China; ^3^ Department of Medicinal Plants, Faculty of Agriculture and Natural Resources Arak University Arak Iran; ^4^ North China Forestry Experiment Center Chinese Academy of Forestry Beijing China

**Keywords:** antiviral defence mechanisms, metabolic reprogramming, metabolism, plant virus, plant–virus interactions

## Abstract

Plant viruses pose a significant threat to global agriculture, leading to substantial crop losses that jeopardise food security and disrupt ecosystem stability. These viral infections often reprogramme plant metabolism, compromising key pathways critical for growth and defence. For instance, infections by cucumber mosaic virus alter amino acid and secondary metabolite biosynthesis, including flavonoid and phenylpropanoid pathways, thereby weakening plant defences. Similarly, tomato bushy stunt virus disrupts lipid metabolism by altering the synthesis and accumulation of sterols and phospholipids, which are essential for viral replication and compromise membrane integrity. Recent advancements in gene‐editing technologies, such as CRISPR/Cas9, and metabolomics offer innovative strategies to mitigate these impacts. Precise genetic modifications can restore or optimise disrupted metabolic pathways, enhancing crop resilience to viral infections. Metabolomics further aids in identifying metabolic biomarkers linked to viral resistance, guiding breeding programmes aimed at developing virus‐resistant plants. By reducing the susceptibility of crops to viral infections, these approaches hold significant potential to reduce dependence on chemical pesticides, increase crop yields and promote sustainable agricultural practices. Future research should focus on expanding our understanding of virus–host interactions at the molecular level while exploring the long‐term ecological impacts of viral infections. Interdisciplinary approaches integrating multi‐omics technologies and sustainable management strategies will be critical in addressing the challenges posed by plant viruses and ensuring global agricultural stability.

## Graphical abstract

This review highlights how plant viruses reprogramme host metabolism, elucidates underlying mechanisms and discusses advanced antiviral strategies, including gene‐editing and metabolomics‐guided approaches.

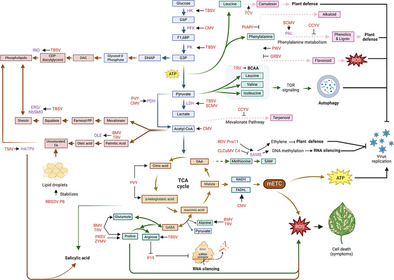

## 1 Introduction

Plant viruses are ubiquitous, affecting a wide range of plant species and exerting profound impacts on global agriculture and ecosystems (Wu et al. 2024; Savary et al. 2019; Mangang et al. 2024). Although the detrimental effects of plant viruses on crop health are well documented, the molecular mechanisms underlying their pathogenesis remain an area of active investigation. In particular, the deep reprogramming of plant metabolism during viral infections is a critical factor in understanding viral pathophysiology (Jiang and Zhou 2023; Wang 2015). Recent studies highlight that plant viruses not only exploit host metabolic resources for their life cycles but also manipulate host metabolic networks to suppress immune responses and facilitate their spread.

During viral infection, the plant metabolic system—encompassing primary metabolism, which is closely tied to energy production and growth, and secondary metabolism, which is pivotal in stress responses—undergoes significant reprogramming to support both viral replication and plant defence mechanisms. For instance, cucumber mosaic virus (CMV) disrupts carbon assimilation, altering sugar distribution, and reallocates sugars to the phloem as an energy source and structural building block for viral replication and systemic movement (Gil et al. 2011; Shalitin and Wolf 2000). Similarly, tomato bushy stunt virus (TBSV) affects lipid metabolism, altering membrane composition to enhance the formation of viral replication sites, thereby promoting efficient viral assembly and spread (Sharma et al. 2010). Beyond primary metabolism, viruses regulate secondary metabolism to reshape host defence mechanisms. For instance, grapevine red blotch virus (GRBV) suppresses the phenylpropanoid pathway and its derivatives, reducing the host plant's ability to produce antiviral compounds (Rumbaugh et al. 2022). Conversely, some studies show that plant viruses modulate phenolic compound production to counteract infection (Rabie et al. 2024; Rashad et al. 2020; Abdelkhalek, Király, et al. 2022). These findings underscore the dynamic and intricate metabolic interactions between viruses and their host plants.

Recent advancements in metabolomics have significantly expanded our understanding of the metabolic changes associated with viral infections. By identifying and quantifying metabolite variations in response to infection, metabolomics not only reveals virus‐specific metabolic reprogramming in plants but also provides critical insights into the biochemical pathways involved in plant defence and stress responses. For example, a study on cucumber plants infected with cucurbit chlorotic yellows virus (CCYV) identified significant alterations in 612 metabolites, linking changes in flavonoid, lipid and amino acid levels to viral replication and symptom development (Zhang et al. 2022). Additionally, by integrating metabolomics with genomics, transcriptomics and proteomics, researchers can unravel the complex biological processes underlying plant–virus interactions, providing a multidimensional understanding of how specific gene expression changes correlate with altered metabolic pathways during infection, ultimately advancing our knowledge of viral pathogenesis and symptom development (Sade et al. 2015; Jiang, Du, Xie, et al. 2023; Siriwan et al. 2023). For example, by integrating transcriptomics and metabolomics, researchers identified the malate circulation pathway as closely linked to the development of mosaic symptoms, shedding light on how metabolic reprogramming during viral infections contributes to symptom manifestation (Jiang, Du, Xie, et al. 2023).

In light of these findings, in‐depth analyses of virus‐induced metabolic reprogramming using metabolomics and multi‐omics approaches provide critical insights for developing virus‐resistant crops. By identifying the key metabolic pathways and regulatory mechanisms involved in plant defence, scientists can leverage precise gene‐editing tools such as CRISPR/Cas9 to modulate these pathways and enhance crop antiviral capacity (Wani et al. 2023; Hinge et al. 2021; Zaidi et al. 2016). For instance, through the editing of multiple metabolic pathway genes in soybean, such as *GmF3H1*, *GmF3H2* and *GmFNSII‐1*, the isoflavone content was significantly increased, which enhanced resistance to soybean mosaic virus (SMV) (Zhang et al. 2020). Furthermore, modulating salicylic acid (SA) and jasmonic acid (JA) signalling pathways and enhancing the synthesis of secondary metabolites, such as phenolics and flavonoids, have been shown to significantly boost plant antiviral defences by improving oxidative stress management and immune responses (Nandy et al. 2021; Thiruvengadam et al. 2016; Woch et al. 2023). These multi‐omics–driven studies not only provide a deeper theoretical understanding of plant antiviral mechanisms but also lay the foundation for applying gene‐editing technologies in agricultural antiviral research.

This article aimed to highlight how plant viruses reprogramme host metabolism and discuss the insights gained from metabolomics into how these metabolic changes influence viral pathogenesis. We will also explore the application potential of multi‐omics approaches in developing strategies for disease‐resistant crops, specifically in combating viral pathogens. These strategies not only enhance resilience against biotic stress, including viral infections, but also reduce reliance on chemical pesticides, contributing to sustainable agricultural development.

## 2 The Role of Host Metabolism in Plant Virus Infection

### 2.1 Carbohydrate and Energy Metabolism

Carbohydrate metabolism is a crucial pathway for producing cellular energy. It converts glucose into ATP through three main processes: glycolysis, the tricarboxylic acid (TCA) cycle and oxidative phosphorylation. In glycolysis, glucose is broken down into pyruvate in the cytoplasm, generating a small amount of ATP and NADH. Next, when pyruvate enters the mitochondria, it enters the TCA cycle, which leads to further oxidation and produces additional NADH, FADH2 and a small amount of ATP. Finally, oxidative phosphorylation takes place in the inner membrane of the mitochondria. Here, NADH and FADH2 assist in synthesising ATP through the electron transport chain, resulting in a significant yield of ATP (Plaxton 1996; Fernie et al. 2004; Dumont and Rivoal 2019).

Viruses, including those that affect plants and humans, often exploit the host's glycolytic pathway to generate the energy needed for their replication. They enhance the expression of glycolytic enzymes, which facilitates the rapid conversion of glucose into pyruvate and increases ATP production. Some viruses even relocate glycolytic enzymes to specific replication sites, boosting the efficiency of ATP production (Nagy et al. 2016; Nagy and Lin 2020; Liu et al. 2024; Chuang et al. 2017; Prasanth et al. 2017). For instance, TBSV takes advantage of the host's glycolytic pathway to produce ATP for its rapid replication (Molho et al. 2021). TBSV may also hijack fermentation pathways, which convert glucose to lactate or ethanol under anaerobic conditions, to quickly regenerate NAD^+^, thus maintaining glycolysis (Lin et al. 2019). Research indicates that sugarcane mosaic virus (SCMV) infection upregulates lactate dehydrogenase (LDH) activity in fermentation pathways, leading to lactate accumulation. This accumulation weakens the plant's defence response and facilitates viral infection. Additionally, viruses may relocate LDH to their replication complexes, further enhancing their replication efficiency (Jiang, Du, Wang, et al. 2023).

Viruses adeptly manipulate host respiratory metabolism to optimise conditions for their replication, often leading to significant alterations in the host's cellular processes. A notable example is the infection of plants by potato virus Y (PVY), where initial stages are marked by a decrease in carbohydrate levels within the leaves, followed by a subsequent increase. This fluctuation extends to intermediates of the γ‐aminobutyric acid shunt and the TCA cycle, suggesting that PVY reprogrammes host respiratory pathways to both facilitate its replication and modulate plant defence mechanisms (Jiang and Zhou 2023; Kogovšek et al. 2016; Osterbaan and Fuchs 2019). Similarly, CMV infection leads to a reduction in soluble sugars and starch in host plants, while concurrently elevating respiration‐related metabolites. This shift indicates a viral strategy to redirect the host's primary metabolic resources towards pathways that favour viral proliferation (Shalitin and Wolf 2000). In the case of SCMV, infection results in the accumulation of glycolytic and C4 dicarboxylic acid pathway intermediates, such as malate. The entry of malate into the mitochondrial TCA cycle activates the mitochondrial electron transport chain, leading to an overproduction of reactive oxygen species (ROS). This oxidative stress manifests as mosaic symptoms in the plant (Jiang, Du, Xie, et al. 2023).

Beyond targeting glycolysis and fermentation pathways, viruses can impair mitochondrial oxidative phosphorylation, diminishing ATP production in the host. TBSV, for instance, reprogrammes host metabolism to favour glycolysis over mitochondrial oxidative phosphorylation, ensuring a rapid ATP supply for viral replication (Nagy and Lin 2020). Early stages of PVY infection are characterised by suppressed host respiration, compromising mitochondrial function and reducing ATP generation via oxidative phosphorylation. Consequently, the host becomes increasingly dependent on glycolysis to meet the energy demands imposed by swift viral replication (Kogovšek et al. 2016). CMV infection similarly disrupts the efficiency of both photosynthesis and mitochondrial oxidative phosphorylation, compelling the host to utilise alternative pathways, such as glycolysis, for ATP production (Tecsi et al. 1996; Fernie et al. 2004).

In summary, viruses orchestrate a complex reprogramming of host respiratory metabolism, strategically redirecting energy production pathways to create a cellular environment conducive to their replication. This manipulation not only facilitates viral proliferation but also intersects with the host's defence responses, underscoring the intricate interplay between plant viruses and their hosts.

### 2.2 Reconfiguration of Lipid Metabolism

Lipids are multifunctional biomolecules that perform essential roles in cellular energy production, signalling pathways and membrane integrity. These functions are particularly significant during plant virus infections, where lipid metabolism is extensively reprogrammed to support viral replication and evasion of host defences (Okazaki and Saito 2014; Hou et al. 2016; Shah 2005). Cellular membranes derived from various organelles provide structural scaffolds for viral replication complexes (VRCs), while simultaneously protecting viral components from RNA‐silencing–based defences and innate immunity mechanisms (Heaton and Randall 2011; Ahlquist et al. 2003). Positive‐strand RNA viruses, in particular, rely heavily on host membrane reorganisation to establish replication factories (Stapleford and Miller 2010; Xu and Nagy 2014).

Recent studies highlight that plant viruses actively disrupt lipid biosynthesis and metabolism, particularly targeting sterols, phospholipids and fatty acids. Such disruptions alter membrane fluidity and plasticity, both of which are critical for the proper assembly and stability of VRCs (Konan and Sanchez‐Felipe 2014; Lorizate and Kräusslich 2011; Stapleford and Miller 2010; de Castro et al. 2016). For example, studies using yeast models demonstrate that inactivating transcriptional activators INO (involved in phospholipid biosynthesis) and ERG (involved in sterol biosynthesis) significantly reduces TBSV replication. Similarly, silencing the sterol biosynthesis homologue *NbSMO* in *Nicotiana benthamiana* decreases TBSV accumulation, confirming the indispensable role of sterols and phospholipids in viral replication (Sharma et al. 2011, 2010).

Fatty acid metabolism has emerged as another critical determinant of virus–host interactions. The enzyme Δ9 fatty acid desaturase, encoded by the *OLE1* gene in yeast, is required for the synthesis of unsaturated fatty acids. A mutation in *OLE1* severely inhibits the replication of brome mosaic virus (BMV), demonstrating the reliance of viruses on unsaturated fatty acids for membrane biogenesis and VRC formation (Lee et al. 2001; Lee and Ahlquist 2003). Similarly, *Arabidopsis fad2* mutants, which have low levels of unsaturated fatty acids, exhibit reduced accumulation of tobacco rattle virus (TRV) (Fernández‐Calvino et al. 2014). This highlights the conservation of lipid requirements across host species and viral families.

In addition to biosynthesis pathways, non‐specific lipid transfer proteins (nsLTPs) play pivotal roles in antiviral defence. In tobacco, the lipid transfer protein NbLTP1 enhances immune responses by increasing SA accumulation and activating NPR1‐mediated downstream signalling, which induces *pathogenesis‐related* (PR) genes. At later stages of infection, NbLTP1 promotes the expression of ROS scavenging genes, which mitigate excessive oxidative stress and preserve membrane integrity (Zhu et al. 2023) This dual action—enhancing defence signalling while maintaining redox balance—underscores the critical role of nsLTPs in coordinating plant antiviral responses.

Lipid droplets (LDs), previously known as lipid storage organelles, have recently been implicated in plant–virus interactions. Plant viruses exploit LD‐associated proteins to regulate fatty acid metabolism, facilitating viral proliferation and symptom development. For instance, in rice infected by rice black‐streaked dwarf virus (RBSDV), the core capsid protein P8 interacts with the LD‐associated protein ZmLDAP2. This interaction stabilises LDs by preventing their degradation, which would otherwise be mediated by the PUX10 protein containing a UBX domain. As a result, LD stabilisation maintains levels of C18 polyunsaturated fatty acids (PUFAs), which are critical for viral replication and systemic spread (Wang et al. 2024). Such interactions reveal the multifunctional roles of LDs beyond simple energy storage, showcasing their importance in viral infection cycles.

In conclusion, plant viruses exploit multiple facets of lipid metabolism, including sterol and fatty acid biosynthesis, nsLTP activity and LD stabilisation, to manipulate host cellular processes. By altering membrane composition, plasticity and signalling pathways, viruses optimise the cellular environment to support their replication. Continued research into the molecular mechanisms of lipid–virus interactions will not only enhance our understanding of viral pathogenesis but also identify novel targets for developing antiviral strategies to safeguard crop productivity.

### 2.3 Amino Acid Metabolism and Protein Synthesis

Plant viruses are obligate parasites that rely entirely on host plants for their replication and systemic spread. Amino acids supplied by the host are crucial not only for viral protein synthesis but also for the production of viral nucleic acids. To meet these demands, viruses reconfigure host metabolic pathways, often causing significant perturbations in amino acid metabolism. Studies show that late stages of infections caused by tobacco mosaic virus (TMV), zucchini yellow mosaic virus (ZYMV), papaya ringspot virus, BMV and TRV are marked by substantial increases in amino acids such as alanine, glutamine and proline. These increases may reflect the need for amino acid–derived metabolites essential for viral protein synthesis (including translation) and nucleic acid synthesis (López‐Gresa et al. 2012; Blua et al. 1994; Wijendra et al. 1995; Xu et al. 2008). Additionally, amino acids like arginine and glutamine are directly implicated in viral replication by serving as precursors for metabolites required for viral genome synthesis and structural protein assembly (Hirabara et al. 2021; Pedrazini et al. 2024; Allen et al. 2022). The association between amino acids and viral spread is linked to host amino acid metabolism, which plays a crucial role in modulating insect vector behaviour, such as aphids and whiteflies, ultimately enhancing virus uptake and transmission efficiency. Studies have shown that CMV infection alters amino acid profiles in host plants, increasing levels of key amino acids like glutamine and proline, which influence aphid feeding preferences. This metabolic shift not only prolongs aphid probing but also accelerates their movement between plants, facilitating more efficient virus spread (Mauck et al. 2014, 2015).

Interestingly, amino acid metabolism is also intricately linked to plant antiviral defence mechanisms. Several studies indicate that certain amino acids accumulate at higher levels in resistant plants compared with susceptible ones (Sade et al. 2015; Kogovšek et al. 2016). For example, in *Arabidopsis*, proline accumulates during the hypersensitive response (HR) to limit viral spread by inducing rapid programmed cell death at infection sites (Deuschle et al. 2004; Fabro et al. 2004). The exogenous application of proline can even trigger HR‐like cell death in the absence of pathogens, suggesting that proline activates defence signalling pathways, particularly proline‐5‐carboxylate metabolism. This metabolic process promotes the accumulation of ROS, which act as critical defence molecules to restrict pathogen proliferation (Monteoliva et al. 2014; Rojas et al. 2014; Qamar et al. 2015). Similarly, arginine plays a defensive role by inhibiting viral proteins through protein arginine methylation. For instance, protein arginine methyltransferase 6 suppresses the activity of the TBSV P19 protein, a viral RNA‐silencing suppressor, thereby enhancing the host's RNA‐silencing–based defence (Zhu et al. 2024).

Paradoxically, amino acid accumulation can also contribute to pathogen susceptibility. For instance, *Arabidopsis lht1* mutants, which exhibit reduced levels of amino acids such as alanine, glutamine and proline, display enhanced resistance to viral and other pathogen infections. Glutamine deficiency in these mutants promotes the accumulation of SA, which activates defence responses, including the expression of *PR‐1* genes and increased ROS production (Liu et al. 2010). Conversely, exogenous application of glutamine suppresses SA accumulation, highlighting glutamine's dual role as both a metabolic resource and a modulator of plant immunity (Rojas et al. 2014). Interestingly, *lht1* mutants also secrete excess glutamine, which may disrupt interactions with beneficial microbes while enhancing resistance against pathogens (Agorsor et al. 2023).

Branched‐chain amino acid (BCAA) metabolism further influences virus–host dynamics. BCAA pathways are linked to plant susceptibility to viruses through their interaction with the target of rapamycin (TOR) signalling pathway, which regulates protein synthesis, growth and autophagy. Disruption of BCAA metabolism affects TOR activity, leading to an imbalance in protein homeostasis and metabolites, which weakens plant defences, particularly during viral infections (Cao et al. 2019). For example, studies show that inactivation of the *DARK INDUCIBLE 4* gene limits TRV proliferation, underscoring the role of BCAA metabolism in viral susceptibility (Fernández‐Calvino et al. 2014).

Genes regulating amino acid metabolism also play crucial roles in viral pathogenesis. One such example is *S*‐adenosyl‐l‐methionine synthetase (SAMS), which catalyses the conversion of l‐methionine and ATP into *S*‐adenosyl‐l‐methionine (SAM), a precursor for ethylene and polyamine biosynthesis. The Pns11 protein of rice dwarf virus (RDV) interacts with OsSAMS1, enhancing its enzymatic activity and increasing ethylene production. Elevated ethylene levels weaken host defences and render rice more susceptible to RDV infection (Zhao et al. 2017). Conversely, viruses such as cotton leaf curl Multan virus (CLCuMV) target SAMS to inhibit its activity, reducing DNA methylation and thereby interfering with transcriptional and post‐transcriptional gene silencing mechanisms. This manipulation enhances viral infectivity and systemic spread (Ismayil et al. 2018).

In summary, viral infections profoundly impact amino acid metabolism to support their replication while also interacting with host defence pathways. Specific amino acids, such as proline and arginine, act as critical regulators of defence responses through ROS production and post‐translational modifications, respectively. Conversely, imbalances in amino acid levels, such as glutamine accumulation, can either enhance susceptibility or trigger plant immunity depending on the context. The dual roles of amino acid metabolism—as a viral target and as a defence regulator—underscore its importance in the complex interplay between viruses and their host plants. Future research focusing on the precise molecular mechanisms underlying amino acid manipulation will provide novel insights for developing virus‐resistant crops and improving plant health.

### 2.4 Alterations in Secondary Metabolites

Secondary metabolites, small organic compounds produced by plants, play pivotal roles in signalling and defence during both biotic and abiotic stresses. These metabolites, including flavonoids, phenolics, terpenoids and alkaloids, are critical components of the plant's immune system. Viral infections often reconfigure secondary metabolic pathways, leading to substantial changes in their accumulation and altering the plant's defence capacity, growth and overall fitness. For instance, in *Arabidopsis thaliana* , infection with turnip crinkle virus (TCV) induces a substantial increase in camalexin, a tryptophan‐derived phytoalexin that enhances defence against pathogens (Dempsey et al. 1997). Genetic variations among *Arabidopsis* ecotypes further demonstrate variability in camalexin accumulation following infections with cauliflower mosaic virus (CaMV), suggesting that genetic factors influence metabolic defence responses (Callaway et al. 1996). This highlights the dynamic relationship between viral infection and host‐specific metabolic regulation.

Similar regulatory phenomena have been observed in cucumbers infected with cucumber chlorotic yellows virus, where secondary metabolites exhibit temporal and spatial regulation. At 7 days post‐infection (dpi), levels of flavonoids, terpenoids and lipid compounds decrease significantly, while amino acids and nucleotide derivatives accumulate, reflecting early viral exploitation of host metabolism. By 15 dpi, lipid accumulation increases further, probably playing a role in viral replication and the plant's response (Zhang et al. 2022). However, the continued decline in flavonoid levels impairs the plant's defence capacity, demonstrating a virus‐driven suppression of key defence metabolites. In *Zea mays* , SCMV infection stimulates the upregulation of *phenylalanine ammonia‐lyase* (*PAL*), a key enzyme in the phenylpropanoid pathway, that regulates SA and lignin biosynthesis. Enhanced PAL expression results in increased SA accumulation, which activates defence‐related genes, while lignin deposition strengthens structural barriers against viral spread (Yuan et al. 2019). This intersection between viral infection and secondary metabolite regulation demonstrates the importance of structural and biochemical defence responses during infection.

The consequences of viral infections on secondary metabolism extend beyond defence responses and have significant economic implications for agricultural crops. In grapevines infected with GRBV, suppression of the phenylpropanoid pathway reduces flavonoid and anthocyanin content, leading to poor wine quality (Blanco‐Ulate et al. 2017). GRBV infections cause uneven ripening, reduced sugar accumulation and increased acidity, which negatively impact grape flavour and aroma (Jelínek et al. 2012). In contrast, infection with grapevine leafroll‐associated virus 3 (GLRaV‐3) in the white grape cultivar Malvasía de Banyalbufar results in elevated levels of flavonols and hydroxycinnamic acid derivatives, potentially reflecting a defensive response to viral stress (Montero et al. 2016). Similarly, in hops ( *Humulus lupulus* ), hop mosaic virus (HpMV), apple mosaic virus (ApMV) and hop latent viroid (HLVd) infections disrupt the biosynthesis of bitter acids, polyphenols and essential oils—key compounds that determine beer flavour—resulting in diminished yield and quality, which highlights the broader economic impact of virus‐induced alterations in secondary metabolism (Pistelli et al. 2018).

The influence of viral infections on secondary metabolism extends to medicinal plants, with significant implications for their therapeutic properties. In *Papaver somniferum* (opium poppy), infection with poppy mosaic virus alters the levels of bioactive alkaloids such as morphine, noscapine and codeine, which are critical for pharmaceutical applications (Zaim et al. 2014). Similarly, infections caused by CMV and passionfruit woodiness virus in passionfruit ( *Passiflora edulis* ) increase the accumulation of phenolics and flavonoids, enhancing antiviral defences but simultaneously altering the plant's medicinal and nutritional properties (Lan et al. 2020). In saffron ( *Crocus sativus* ), saffron latent virus (SaLV) infections significantly influence the biosynthesis of crocins and kaempferol, key metabolites responsible for saffron's colour, aroma and medicinal properties. Alterations in their proportions compromise saffron quality, further underscoring the detrimental effects of viral infections on economically and therapeutically valuable crops (Parizad et al. 2019).

The observed metabolic shifts during viral infections reflect the complex interplay between viral exploitation and plant defence. Viral suppression of secondary metabolite pathways, such as flavonoid and phenolic biosynthesis, can impair the plant's ability to produce key defence compounds, weakening its resistance. Conversely, some viruses trigger the upregulation of secondary metabolites, such as SA, lignin and phenolics, as part of the plant's stress response. The balance between metabolic activation and suppression depends on factors such as viral species, host genetics and environmental conditions. For instance, increased lipid accumulation in viral infections, as seen in cucumbers, suggests that lipids may be co‐opted for viral replication or for signalling plant stress responses. Meanwhile, declines in flavonoids and terpenoids reflect virus‐mediated suppression of metabolic pathways essential for plant immunity.

**FIGURE 1:**
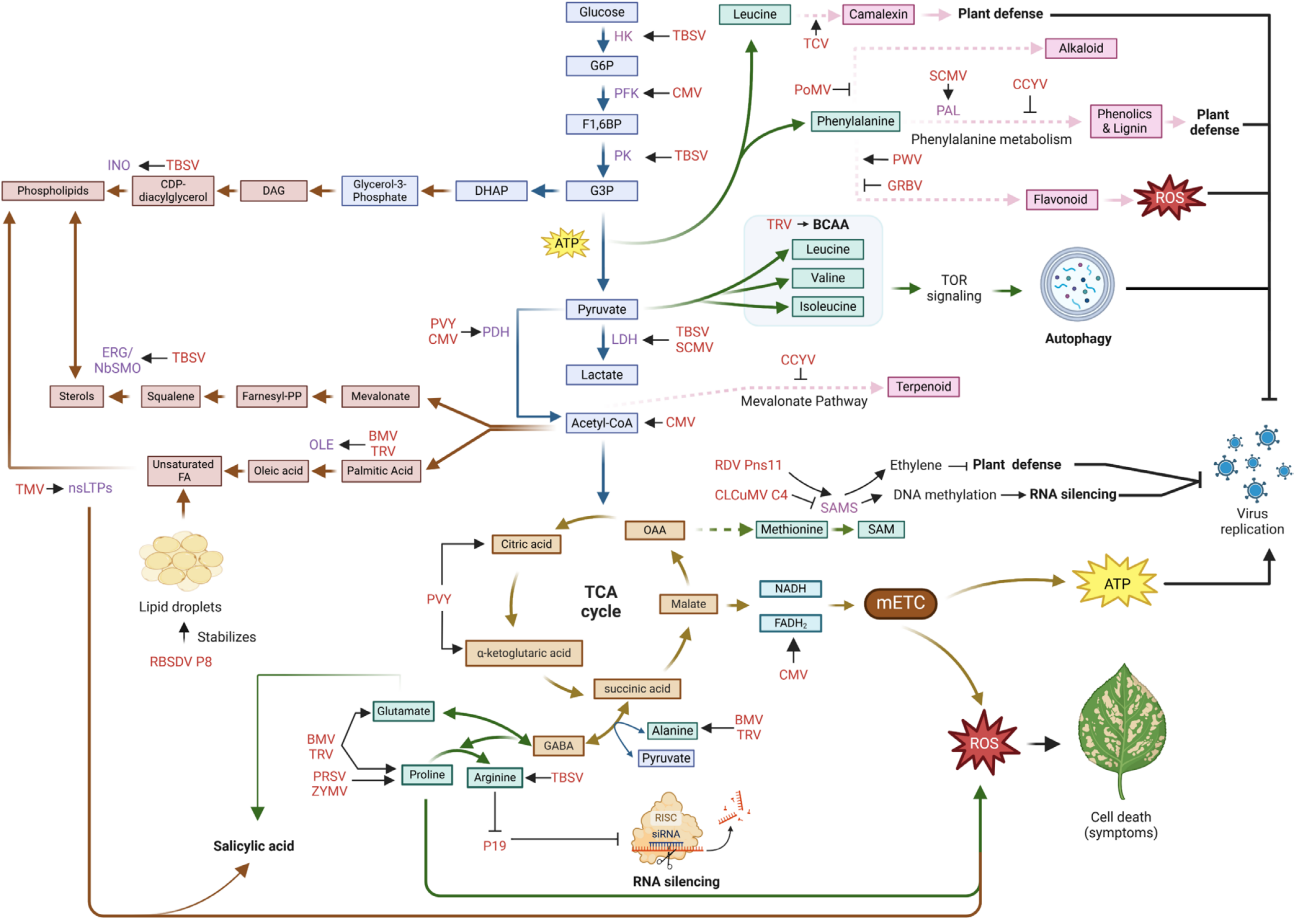
Impacts of viral infection on plant metabolic pathways. This diagram illustrates the interplay between plant metabolism and viral infections, focusing on primary and secondary metabolic pathways and their alterations in response to viral infection. The relationships between metabolic pathways and their respective viral effects or defence mechanisms are highlighted. BCAAs, branched‐chain amino acids; BMV, brome mosaic virus; CCYV, cucurbit chlorotic yellows virus; CLCuMV, cotton leaf curl Multan virus; CMV, cucumber mosaic virus; DAG, diacylglycerol; DHAP, dihydroxyacetone phosphate; F1,6BP, fructose‐1,6‐bisphosphate; FA, fatty acid; Farnesyl‐PP, farnesyl pyrophosphate; G3P, glyceraldehyde‐3‐phosphate; G6P, glucose‐6‐phosphate; GABA, γ‐aminobutyric acid; GRBV, grapevine red blotch virus; HK, hexokinase; LDH, lactate dehydrogenase; mETC, mitochondrial electron transport chain; OAA, oxaloacetate; PAL, phenylalanine ammonia‐lyase; PDH, pyruvate dehydrogenase; PFK, phosphofructokinase; PK, pyruvate kinase; PMV, poppy mosaic virus; PRSV, papaya ringspot virus. PVY, potato virus Y; PWV, passionfruit woodiness virus; RBSDV, rice black‐streaked dwarf virus; RDV, rice dwarf virus; ROS, reactive oxygen species; SAM, *S*‐adenosyl methionine; SSAMS, S‐adenosyl‐l‐methionine synthetase; SCMV, sugarcane mosaic virus; TBSV, tomato bushy stunt virus; TCA, tricarboxylic acid; TCV, turnip crinkle virus; TMV, tobacco mosaic virus; TRV, tobacco rattle virus; Unsaturated FA, unsaturated fatty acids; ZYMV, zucchini yellow mosaic virus.

Viral infections significantly alter plant secondary metabolism, influencing defence signalling, structural integrity and crop quality. These metabolic changes can either enhance or reduce host resistance, ultimately affecting ecological roles and economic outcomes. Understanding the molecular mechanisms underlying these alterations is crucial for developing virus‐resistant crops, optimising secondary metabolite production and ensuring the quality of agricultural and medicinal plants. Future research should focus on integrating multi‐omics approaches to uncover the dynamic regulation of secondary metabolites during viral infections, enabling targeted strategies to mitigate the impacts of plant viruses on global agriculture (Figure 1, Table 1).

**TABLE 1:** Summary of virus‐induced metabolic reprogramming in plants: Pathways, metabolites and key findings.

Plant species	Metabolite types	Virus	Infection type	Pathways explored	Key findings	References
Melon ( *Cucumis melo* )	Flavonoids, lipids, amino acids	CMV	Systemic	Carbon assimilation, sugar redistribution	CMV reallocates sugars to the phloem as an energy source and structural building block for replication and movement	Gil et al. 2011; Shalitin and Wolf 2000
*Nicotiana benthamiana*	Carbohydrates, ATP, NADH, FADH2	TBSV	Systemic	Glycolysis, fermentation, oxidative phosphorylation	TBSV hijacks glycolysis and fermentation pathways to increase ATP production, enhancing viral replication	Nagy et al. 2016; Molho et al. 2021; Lin et al. 2019
Maize ( *Zea mays* )	Glycolytic intermediates, malate	SCMV	Systemic	Glycolysis, C4 dicarboxylic acid pathway, mitochondrial TCA cycle	SCMV infection leads to malate accumulation, mitochondrial reactive oxygen species (ROS) overproduction and mosaic symptoms	Jiang, Du, Xie, et al. 2023
Maize ( *Z. mays* )	NADH, lactate	SCMV	Systemic	Fermentation, lactate dehydrogenase activity	SCMV upregulates LDH activity, leading to lactate accumulation, weakening plant defence and enhancing viral infection	Jiang, Du, Wang, et al. 2023
Potato ( *Solanum tuberosum* )	Carbohydrates, TCA intermediates	PVY	Systemic	Glycolysis, TCA cycle, GABA shunt	PVY infection reprogrammes carbohydrate metabolism, facilitating replication and modulating defence responses	Kogovšek et al. 2016
Melon ( *C. melo* )	Soluble sugars, starch, respiration‐related metabolites	CMV	Systemic	Carbon assimilation, glycolysis, oxidative phosphorylation	CMV reduces sugars and starch, elevates respiration‐related metabolites and redirects resources for viral proliferation	Shalitin and Wolf 2000
*N. benthamiana*	ATP, NADH, FADH2	TBSV	Systemic	Oxidative phosphorylation, glycolysis	TBSV reprogrammes host metabolism, favouring glycolysis over oxidative phosphorylation for ATP supply	Nagy and Lin 2020
Squash ( *Cucurbita pepo* )	Carbohydrates	CMV	Systemic	Photosynthesis, glycolysis, oxidative phosphorylation	CMV disrupts photosynthesis and mitochondrial oxidative phosphorylation, forcing the host to rely on glycolysis for ATP	Tecsi et al. 1996
Potato ( *S. tuberosum* )	Carbohydrates, ATP, NADH, FADH2	PVY	Systemic	Glycolysis, TCA cycle, oxidative phosphorylation	PVY infection suppresses host respiration, compromising mitochondrial function and reducing ATP generation via oxidative phosphorylation. Consequently, the host becomes increasingly dependent on glycolysis to meet energy demands imposed by rapid viral replication	Kogovšek et al. 2016
*N. benthamiana*	Sterols, phospholipids	TBSV	Experimental inoculation	Lipid biosynthesis	Alters membrane composition to enhance viral replication sites, promoting efficient assembly and spread	Sharma et al. 2010; Sharma et al. 2011
Yeast ( *Saccharomyces cerevisiae* )	Unsaturated fatty acids	BMV	Experimental inoculation	Fatty acid biosynthesis	Mutation in OLE1 inhibited BMV replication, showing reliance on unsaturated fatty acids	Lee et al. 2001; Lee and Ahlquist 2003
*Arabidopsis thaliana*	Unsaturated fatty acids	TRV	Mutant study	Fatty acid biosynthesis	*fad2* mutants exhibited reduced TRV accumulation due to low unsaturated fatty acid levels	Fernández‐Calvino et al. 2014
Tobacco ( *Nicotiana tabacum* )	Non‐specific lipid transfer	TMV	Systemic	Lipid transfer proteins (nsLTPs)	NbLTP1 enhances SA accumulation and ROS scavenging, improving immune responses	Zhu et al. 2023
Maize ( *Z. mays* )	C18 polyunsaturated fatty acids	RBSDV	Systemic	Lipid droplet stabilisation	Interaction between P8 and ZmLDAP2 stabilises lipid droplets for viral replication	Wang et al. 2024
Tomato ( *Solanum lycopersicum* )	Arginine	TBSV	Systemic	Protein arginine methylation	PRMT6 suppresses TBSV P19 activity, enhancing RNA‐silencing–based defence	Zhu et al. 2024
*A. thaliana*	Branched‐chain amino acids (BCAAs)	TRV	Mutant study	BCAA metabolism	Disruption of *DARK INDUCIBLE 4* gene limits TRV proliferation by affecting BCAA metabolism	Fernández‐Calvino et al. 2014
Rice ( *Oryza sativa* )	*S*‐adenosyl‐l‐methionine (SAM)	RDV	Systemic	SAM metabolism and ethylene biosynthesis	Pns11 enhances SAMS activity, increasing ethylene production and reducing host resistance	Zhao et al. 2017
Tobacco ( *N. tabacum* )	SAM	CLCuMV	Systemic	DNA methylation	Virus targets SAM to inhibit DNA methylation, suppressing host silencing mechanisms	Ismayil et al. 2018
Tomato ( *S. lycopersicum* )	Amino acids, sugars, organic acids	TMV	Systemic	Phenylpropanoid, flavonoid biosynthesis	Flavonoids accumulate in infected leaves and phenylpropanoids in systemic leaves. Sugars and organic acids vary with time and leaf position	López‐Gresa et al. 2012
Squash ( *C. pepo* )	Amino acids, sugars, starch	ZYMV	Systemic	Amino acid and sugar metabolism	ZYMV infection increases amino acids in leaves and alters phloem amino acid composition. Starch content decreases over time in infected plants	Blua et al. 1994
*A. thaliana*	Camalexin	TCV	Systemic	Tryptophan‐derived phytoalexin pathway	Induces substantial increase in camalexin, enhancing defence against pathogens	Dempsey et al. 1997
*A. thaliana*	Camalexin	CaMV	Systemic	Camalexin biosynthesis	Variability in camalexin accumulation across ecotypes shows genetic influence on metabolic defence responses	Callaway et al. 1996
Cucumber ( *Cucumis sativus* )	Flavonoids, lipids, amino acids	CCYV	Localised, systemic	Lipid metabolism, flavonoid biosynthesis	Early reduction in flavonoids and terpenoids; increased lipid accumulation at later stages supports viral replication	Zhang et al. 2022
Maize ( *Z. mays* )	Salicylic acid (SA), lignin	SCMV	Systemic	Phenylpropanoid pathway	Upregulation of phenylalanine ammonia‐lyase (PAL) enhances SA and lignin synthesis, activating defence genes and strengthening structural barriers	Yuan et al. 2019
Grapevine ( *Vitis vinifera* )	Flavonoids, anthocyanins	GRBV	Systemic	Phenylpropanoid pathway suppression	Suppresses flavonoid and anthocyanin synthesis, reducing grape quality (e.g., uneven ripening, lower sugar levels and increased acidity)	Jelínek et al. 2012; Blanco‐Ulate et al. 2017
Grapevine ( *V. vinifera* )	Flavonols, hydroxycinnamic acids	GLRaV‐3	Systemic	Phenolic biosynthesis	Increased flavonol and hydroxycinnamic acid derivatives, possibly a defensive response to viral stress	Montero et al. 2016
Hop ( *Humulus lupulus* )	Bitter acids, polyphenols, essential oils	HpMV, ApMV, HLVd	Systemic	Secondary metabolite biosynthesis	Viral infections disrupt secondary metabolite biosynthesis, affecting flavour and reducing yield and quality	Pistelli et al. 2018
Opium poppy ( *Papaver somniferum* )	Morphine, noscapine, codeine	PMV	Systemic	Alkaloid biosynthesis	Alters levels of bioactive alkaloids, impacting pharmaceutical properties	Zaim et al. 2014
Passionfruit ( *Passiflora edulis* )	Phenolics, flavonoids	CMV, PMV	Systemic	Phenolic and flavonoid biosynthesis	Increases phenolics and flavonoids, enhancing antiviral defences but altering medicinal and nutritional properties	Lan et al. 2020
Saffron ( *Crocus sativus* )	Crocins, kaempferol	SaLV	Systemic	Carotenoid and flavonoid biosynthesis	Alters crocin and kaempferol levels, compromising saffron quality in terms of colour, aroma and medicinal properties	Parizad et al. 2019
Grapevine ( *V. vinifera* )	Phenylpropanoids, secondary metabolites	GRBV	Systemic	Phenylpropanoid pathway	Suppresses phenylpropanoid pathway, reducing antiviral compound production and compromising defence	Rumbaugh et al. 2022

## 3 Application of Metabolic Regulation in Antiviral Strategies

### 3.1 Metabolic Engineering Strategies: Antiviral Plant Breeding Through Metabolic Reprogramming

Metabolic reprogramming represents a promising strategy for developing virus‐resistant crops by regulating secondary metabolic pathways to enhance disease resistance. Gene‐editing tools, such as CRISPR/Cas9, enable precise manipulation of plant metabolic pathways, facilitating the synthesis of antiviral compounds. For instance, in soybean, CRISPR/Cas9‐mediated multiplex gene editing targeted three key genes involved in flavonoid biosynthesis—*GmF3H1*, *GmF3H2* and GmFNSII‐1. This modification significantly increased the isoflavone content in soybean leaves, thereby enhancing resistance to SMV. Studies demonstrated that elevated isoflavone levels effectively reduced viral protein accumulation, providing a robust metabolic engineering approach for virus‐resistant soybean breeding (Zhang et al. 2020).

Moreover, CRISPR/Cas9 technology has also been employed to regulate hormone metabolism‐related genes to improve antiviral resistance in plants. In rice, knocking out the *vacuolar ATPase subunit D* (*OsV‐ATPase D*) led to a significant increase in JA and abscisic acid levels, conferring enhanced resistance to southern rice black‐streaked dwarf virus. However, this modification simultaneously reduced rice resistance to rice stripe virus, revealing the specificity and complexity of metabolic reprogramming in modulating antiviral defences (Lu et al. 2023).

Further advancements in CRISPR technology have enabled multiplex gene editing, allowing simultaneous regulation of multiple metabolic pathways to optimise plant defence mechanisms (Cao et al. 2020; Mipeshwaree Devi et al. 2023). The ability to edit multiple targets and fine‐tune defence pathways underscores the extensive potential of CRISPR/Cas9 for achieving antiviral crop breeding. Despite the immense promise of these strategies, CRISPR technology still faces challenges, including precision and off‐target effects, particularly in complex genomic contexts. Future efforts must focus on improving high‐fidelity CRISPR tools and enhancing gene delivery systems to overcome these technical hurdles. Nevertheless, integrating gene editing with metabolic engineering offers a transformative approach to enhancing plant antiviral capabilities, presenting innovative solutions for sustainable agriculture on a global scale.

### 3.2 Plant–Microbe Interactions and Metabolic Regulation

Beneficial microbes, such as plant growth‐promoting rhizobacteria (PGPR) and endophytic fungi, play a critical role in regulating plant metabolic pathways and enhancing antiviral defence mechanisms. These microorganisms activate induced systemic resistance (ISR) in plants, stimulating the synthesis of antiviral compounds and significantly strengthening plant immunity.

PGPR, such as *Pseudomonas fluorescens* and *Bacillus subtilis* , cause ISR through JA and ethylene signalling pathways, independent of the SA signalling pathway. This activation triggers broad‐spectrum immune responses in plants, improving resistance to viruses and other pathogens (Annapurna et al. 2013; Meena et al. 2020; Sofy et al. 2019). For example, studies have shown that *B. subtilis* significantly reduces the severity of pepper mild mottle virus infection in pepper plants by modulating JA‐ and ethylene‐dependent pathways (Ahn et al. 2002; Lee and Ryu 2016). Similarly, PGPR have demonstrated effectiveness in controlling CMV in tomato plants, further underscoring their role in antiviral defence (Dashti et al. 2012).

Endophytic fungi, such as *Trichoderma* spp., form symbiotic relationships with plants, activating ISR and regulating the synthesis of key metabolites to enhance plant resistance to viruses. These fungi can also produce bioactive compounds analogous to plant defence molecules, thereby strengthening the host plant's immune system. For instance, certain *Trichoderma* strains generate secondary metabolites with inhibitory activity against viral replication, providing substantial support for plant antiviral defences (Abdelkhalek, Al‐Askar, et al. 2022; Tamandegani et al. 2021).

Additionally, some beneficial microbes release volatile organic compounds (VOCs) that not only directly inhibit pathogen growth but also activate plant metabolic pathways, promoting the synthesis of antiviral metabolites such as flavonoids and phenolic compounds. For example, VOCs produced by *B. subtilis* and *P. fluorescens* disrupt the cellular structure and function of pathogens. Simultaneously, these VOCs act as signalling molecules to activate the plant's immune response, forming a chemical barrier and enhancing overall antiviral capacity (Kanchiswamy et al. 2015; Almeida et al. 2023; Tsai et al. 2023).

Beneficial microbes also indirectly improve plant immunity by optimising the rhizosphere environment and promoting nutrient uptake. PGPR enhance nutrient availability through nitrogen fixation and phosphate solubilisation, thereby improving plant growth and stress tolerance. Furthermore, interactions between these microbes and plant root systems help plants adapt to environmental stresses, reducing dependency on chemical pesticides while fostering sustainable growth (Vlot and Rosenkranz 2022).

The interactions between plants and beneficial microbes involve multiple mechanisms, including ISR activation, antiviral metabolite production, VOC regulation and rhizosphere improvement. These pathways collectively enhance plant antiviral defence capacity. Understanding the molecular mechanisms underlying plant–microbe interactions will provide a solid theoretical foundation for developing sustainable antiviral strategies, reducing the use of chemical pesticides and advancing environmentally friendly agricultural practices.

### 3.3 Natural Compounds and Metabolic Regulation

Natural compounds play a central role in regulating plant metabolism and enhancing antiviral defence mechanisms. These compounds, derived from plant secondary metabolic pathways, include phenolics, flavonoids, alkaloids and VOCs. They not only inhibit the spread of plant viruses but also provide critical support for sustainable agricultural practices.

Phenolic compounds, such as lignin and its derivatives, are key components of plant immune defence systems. They primarily function by strengthening the mechanical integrity of the cell wall, forming a physical barrier that prevents viral invasion and spread. For example, viral infection can upregulate the expression of PAL, promoting lignin biosynthesis and enhancing plant resistance to viral pathogens. Studies have shown that lignin deposition effectively reduces viral accumulation and improves defence against pathogens like SCMV (Yuan et al. 2019).

Flavonoids possess strong antioxidant properties and can inhibit viral replication and spread through both direct and indirect mechanisms. Compounds such as quercetin and kaempferol exhibit significant antiviral activity by interfering with viral RNA synthesis and assembly (Zaynab et al. 2018; Ahmad et al. 2024). Furthermore, flavonoids mitigate oxidative stress induced by viral infections, maintaining cellular homeostasis and enhancing overall plant immunity (Di Ferdinando et al. 2012).

Alkaloids are essential components of plant secondary metabolism and bolster systemic antiviral defence through multilayered mechanisms. For instance, morphine and codeine, alkaloids derived from poppy plants, regulate immune signalling pathways, amplifying defence cascades that significantly suppress viral replication and spread (Ali et al. 2019; Zhang et al. 2023). These alkaloids enhance plant self‐defence by activating the expression of antiviral proteins, thereby strengthening the immune response.

VOCs, such as coumarins, terpenes and aldehydes, exhibit dual modes of defence in plant–virus interactions. On one hand, VOCs inhibit viral infection and replication by disrupting viral protein structures or interfering with viral RNA functionality (Dorokhov et al. 2014). On the other hand, VOCs modulate the rhizosphere microbial community, attracting beneficial microbes while repelling virus‐carrying herbivores. For example, coumarins and terpenes can significantly alter rhizosphere microbial composition, promoting the growth of symbiotic microbes that further induce systemic resistance in plants and enhance overall immunity (Zhao et al. 2022; Ahmad et al. 2024).

The use of natural compounds to strengthen plant antiviral defences provides a sustainable alternative to synthetic chemical pesticides. As core components of biopesticides, phenolics, flavonoids, alkaloids and VOCs reduce reliance on synthetic chemicals, mitigating their environmental impact and contributing to ecosystem health. Moreover, these natural compounds act synergistically with plant immune systems, improving adaptability to emerging viral threats and positioning themselves as integral components of Integrated Pest Management (IPM) strategies.

**FIGURE 2:**
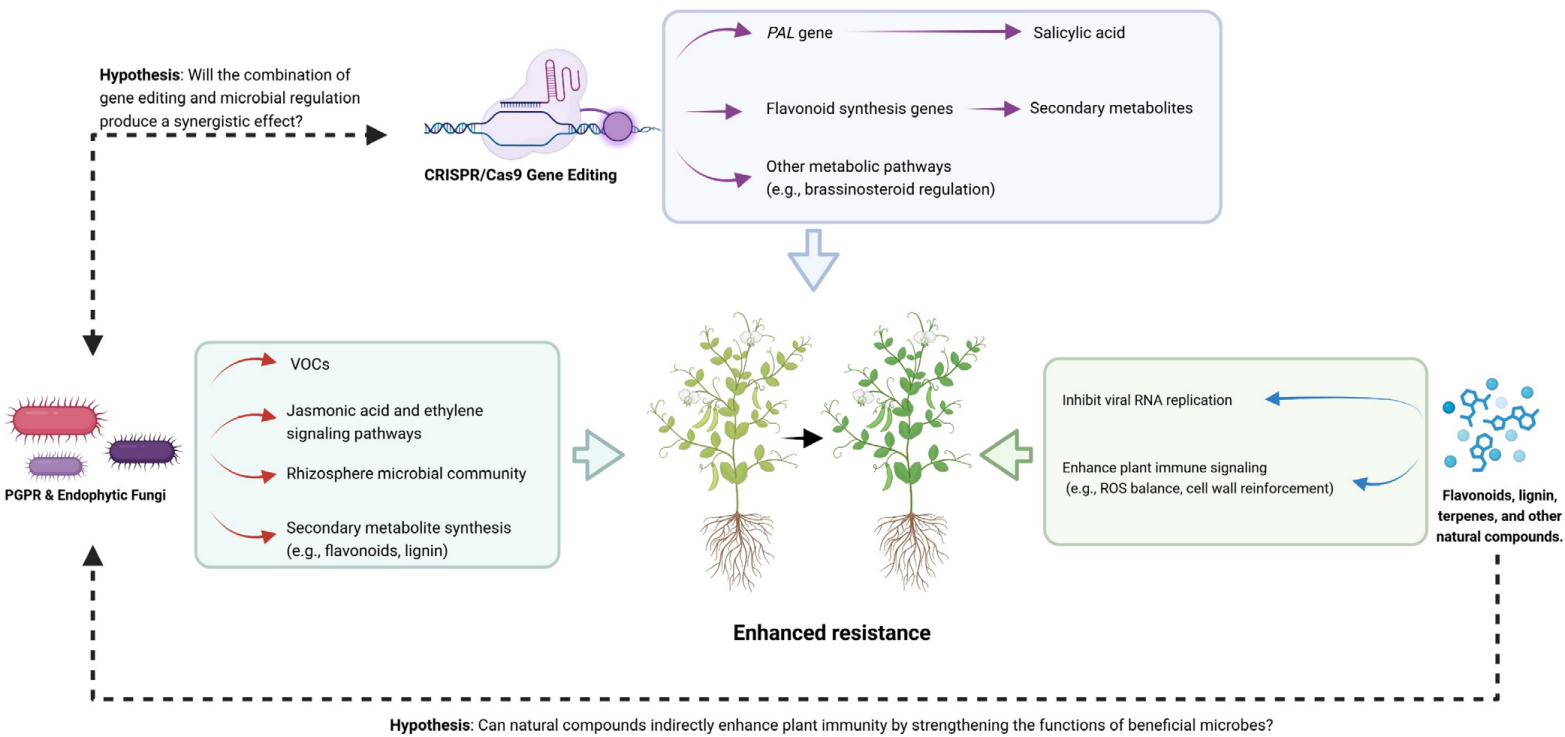
Synergistic strategies for enhancing plant resistance against viral infections. The figure illustrates potential pathways and hypotheses linking CRISPR/Cas9 gene editing, plant growth‐promoting rhizobacteria (PGPR), endophytic fungi and natural compounds to improved plant immunity. The CRISPR/Cas9 gene‐editing approach targets genes involved in salicylic acid synthesis (e.g., *PAL*), flavonoid biosynthesis and other metabolic pathways (e.g., brassinosteroid regulation), leading to enhanced production of secondary metabolites. PGPR and endophytic fungi influence plant immunity through mechanisms such as volatile organic compounds (VOCs), jasmonic acid and ethylene signalling pathways, modulation of the rhizosphere microbial community and stimulation of secondary metabolite synthesis (e.g., flavonoids and lignin). Natural compounds, such as flavonoids, lignin and terpenes, contribute directly by inhibiting viral RNA replication and enhancing plant immune signalling (e.g., reactive oxygen species [ROS] balance and cell wall reinforcement). Hypotheses propose that integrating these strategies—such as combining gene editing and microbial regulation or leveraging natural compounds to strengthen microbial functions—can generate synergistic effects, collectively enhancing resistance against plant viral infections.

In conclusion, integrating metabolic regulation strategies—through genetic engineering, beneficial microbes and natural compounds—offers significant potential for enhancing antiviral defences in plants. This holistic approach not only improves crop resilience against viral infections but also promotes sustainable agricultural practices by reducing reliance on chemical pesticides. As research progresses, these strategies will be vital for ensuring food security and ecosystem health in the face of emerging viral threats (Figure 2).

## 4 Future Research Directions and Challenges

**FIGURE 3:**
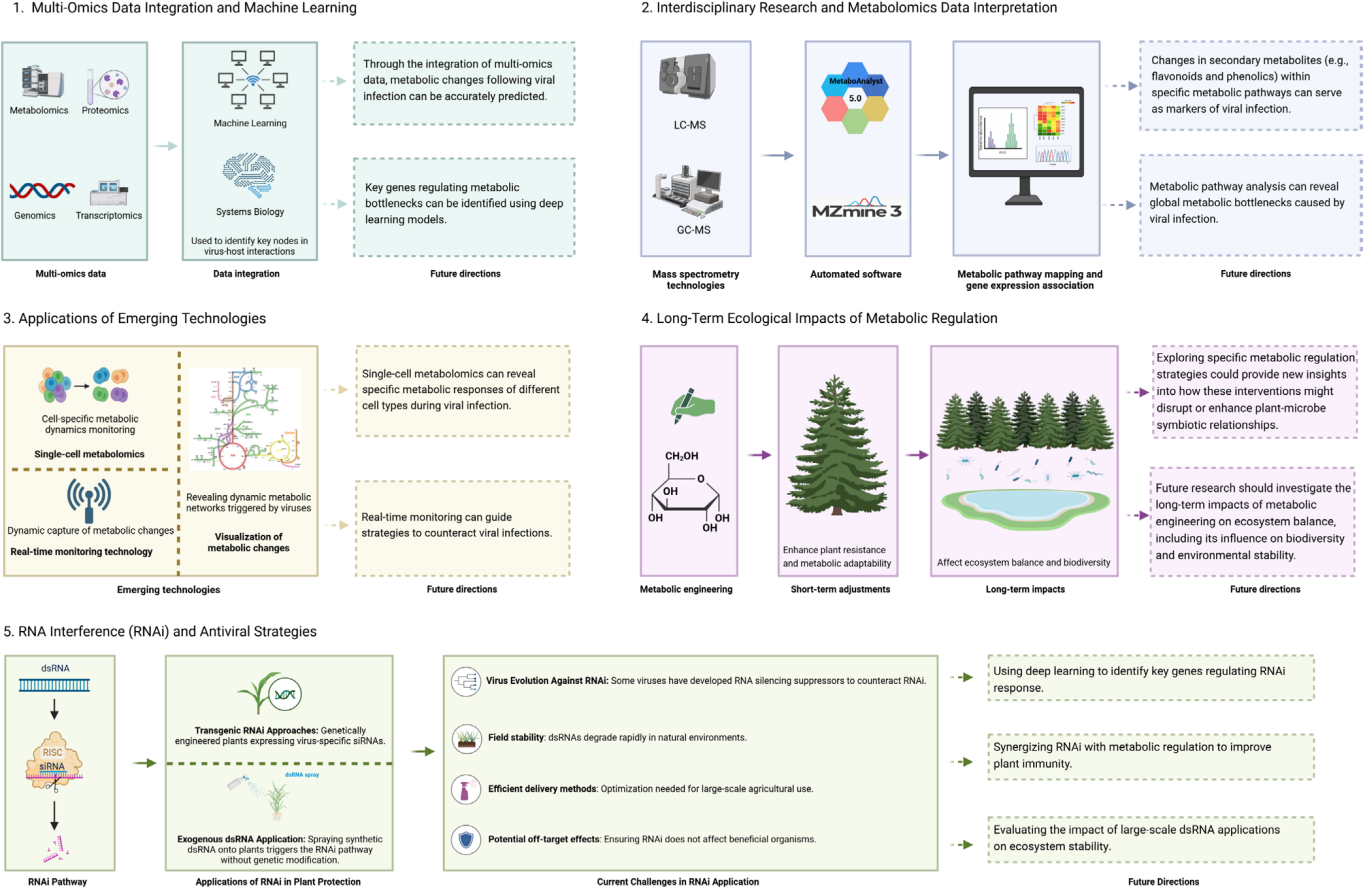
Conceptual framework for future research directions in plant–virus interactions and metabolic regulation. This figure presents key research areas for advancing the understanding of plant–virus interactions and metabolic regulation. (1) Multi‐omics Data Integration and Machine Learning: This section highlights the integration of genomics, transcriptomics, proteomics and metabolomics data through machine learning and systems biology approaches. The goal is to identify key genes and metabolic bottlenecks involved in virus–host interactions. Future directions include utilising deep learning models to predict viral infection‐induced metabolic changes. (2) Interdisciplinary Research and Metabolomics Data Interpretation: Mass spectrometry technologies (LC–MS and GC–MS) are central to metabolomics data collection. The automated software tools (e.g., MZmine 3 and MetaboAnalyst) facilitate metabolic pathway mapping and gene expression analysis. By identifying changes in secondary metabolites (e.g., flavonoids, phenolics), this approach may serve as markers for viral infection, providing valuable insights into metabolic disruptions caused by viral infections. (3) Applications of Emerging Technologies: The application of single‐cell metabolomics enables the study of specific metabolic responses in different plant cell types during viral infection. Real‐time monitoring technologies offer a dynamic capture of metabolic changes, which could guide strategies to counteract viral infections. These emerging technologies promise to deepen our understanding of plant immune responses and metabolic regulation during viral challenges. (4) Long‐Term Ecological Impacts of Metabolic Regulation: This section explores the ecological consequences of metabolic engineering, which can enhance plant resistance and metabolic adaptability. In the long term, such strategies may affect ecosystem balance and biodiversity, potentially disrupting or enhancing plant–microbe symbiotic relationships. Future research should focus on understanding these long‐term impacts and their influence on environmental stability and ecosystem dynamics. (5) RNA Interference (RNAi) and Antiviral Strategies: RNAi strategies can protect plants from viral infections through the silencing of viral genes. Genetically engineered plants expressing virus‐specific small interfering RNAs (siRNAs) can be sprayed onto plants, enhancing the RNA pathway and providing genetic modification for antiviral protection. Current challenges include addressing viral evolution against RNAi, ensuring field stability of dsRNAs in natural environments, optimising delivery methods and minimising off‐target effects. Future directions suggest using deep learning to identify key genes regulating RNAi response, synergizing RNAi with metabolic regulation and assessing the ecological impacts of large‐scale dsRNA applications on ecosystem stability.

Future antiviral strategies encounter a variety of challenges and opportunities, especially in areas such as multi‐omics data integration, machine learning, metabolomics data interpretation and the use of emerging technologies. These fields are advancing rapidly in antiviral development and have significant implications for ecological adaptability and biodiversity in the future (Figure 3).

### 4.1 Multi‐Omics Data Integration and Machine Learning

The future of antiviral research increasingly relies on the integration of multi‐omics data, including genomics, transcriptomics, proteomics and metabolomics, to decipher the complex molecular mechanisms underlying virus–host interactions. These high‐throughput datasets not only provide a comprehensive view of viral pathogenicity and host defence responses but also aid in identifying critical regulatory pathways and biomarkers. For example, combined transcriptomic and metabolomic analyses in plants infected with tomato yellow leaf curl virus revealed significant upregulation of the phenylpropanoid and ureide/polyamine pathways, leading to the accumulation of flavonoids and lignin, thereby enhancing antiviral defence (Sade et al. 2015).

Similarly, proteomic and metabolomic studies of cassava infected with Sri Lankan cassava mosaic virus demonstrated the central role of chlorogenic acid and caffeic acid in ROS regulation and defence pathways. The interaction of protein phosphatase 2C with SA and JA signalling further amplified the host immune response (Siriwan et al. 2023).

Although multi‐omics data integration has driven significant progress in antiviral research, challenges such as data heterogeneity, standardisation issues and computational bottlenecks remain. Machine learning algorithms, including random forests, support vector machines (SVMs) and deep learning models, have emerged as powerful tools for analysing high‐dimensional, complex datasets. These models can identify nonlinear relationships, uncover hidden patterns and efficiently predict novel antiviral targets (Jin et al. 2021; Cao et al. 2018). For instance, deep learning models construct multi‐layer biological networks by integrating transcriptomic and metabolomic data, revealing key regulatory genes and pathways involved in virus–host interactions (Bakker et al. 2018). Tools such as MiBiOmics enable modular network analysis that links multi‐omics data with external phenotypic traits, facilitating the understanding of metabolic adaptations in plants under viral stress (Zoppi et al. 2021).

The integration of multi‐omics data and machine learning presents new opportunities for plant antiviral research, but challenges remain regarding data heterogeneity, limited integration depth and computational optimisation. By introducing spatiotemporal dynamic analysis techniques and metabolic engineering strategies, researchers can systematically decipher plant antiviral mechanisms and bridge fundamental knowledge with applied solutions. This interdisciplinary framework holds promise for achieving precise regulation of plant–virus interactions and advancing crop resistance improvement. Ultimately, it will contribute to sustainable agricultural development by offering forward‐looking solutions for mitigating viral threats.

### 4.2 Interdisciplinary Research and Metabolomics Data Interpretation

The rapid development of metabolomics, particularly high‐resolution platforms such as liquid chromatography–mass spectrometry (LC‐MS) and gas chromatography–mass spectrometry (GC‐MS), enables researchers to capture dynamic metabolic changes in plants under stress conditions. However, fully unlocking the biological significance of these data requires addressing several critical challenges and research directions.

The plant metabolome is highly complex and dynamic, consisting of thousands of metabolites with diverse chemical properties and concentrations. This heterogeneity presents significant challenges in data standardisation and integration. Although tools such as MetaboAnalyst and XCMS are widely used for high‐throughput data processing, the field still lacks unified data standards and annotation frameworks (Pang et al. 2024; Cambiaghi et al. 2017). Future research must focus on developing more efficient and scalable informatics pipelines that integrate artificial intelligence and automated workflows to enable cross‐laboratory data sharing and standardised interpretation.

Currently, most metabolomics studies focus on static data analysis, which fails to capture the spatiotemporal dynamics of virus infections and plant responses. The integration of single‐cell metabolomics and spatial metabolite imaging will allow researchers to track metabolic network changes at the single‐cell or tissue level in real time. These advancements will uncover the precise temporal and spatial interactions between viruses and hosts, providing actionable regulatory targets (Hu et al. 2023; Gilmore et al. 2019).

On the computational front, current bioinformatics tools face limitations in handling large‐scale, multidimensional datasets, particularly in deciphering nonlinear and noisy data. Future progress will depend on the development of smarter, more interpretable machine learning algorithms and network modelling tools. For example, deep learning models can optimise metabolic pathway predictions to identify key regulatory nodes. Integrating metabolic flux analysis with computational models will further elucidate metabolite flow and resource allocation under stress conditions.

### 4.3 Long‐Term Ecological Impacts of Metabolic Regulation

Plant metabolic regulation plays a pivotal role in shaping ecological adaptability and maintaining biodiversity. Although enhancing plant stress resistance through metabolic engineering offers promising results, it may lead to unforeseen ecological consequences. Future studies must address the following critical challenges.

Metabolic engineering that enhances specific traits, such as secondary metabolite accumulation or lignin synthesis, may disrupt plant interactions with other organisms. For instance, high lignin content can reduce plant residue decomposition rates, thereby impacting soil nutrient cycling and fertility (Stewart et al. 2015; Ntonta et al. 2024). Enhanced production of secondary metabolites may also negatively affect the behaviour and population dynamics of herbivores, pollinators and related species (Kariñho‐Betancourt 2018; Kessler and Kalske 2018).

Moreover, metabolic regulation affects critical ecosystem services, including nutrient cycling, carbon fixation, soil health and pollination networks. Optimising nutrient‐use efficiency through metabolic engineering may alter soil nutrient availability, disrupting coexisting species that rely on shared resources (Shukla et al. 2021; Sentenac et al. 2022; Baghalian et al. 2014).

Excessive allocation of resources to specific defence pathways, such as SA or JA signalling, may limit plant growth and reproduction, reducing their overall adaptability to fluctuating environments. Specific metabolic responses to particular stresses may also interfere with broader regulatory networks, impairing the plant's ability to respond to simultaneous or sequential stresses, such as drought and pathogen attack (Karasov et al. 2017).

In conclusion, although metabolic regulation and engineering provide powerful tools for improving plant stress tolerance and productivity, their long‐term ecological impacts must not be overlooked. Future research integrating ecological principles, systems biology and computational modelling will be critical for evaluating the effects of metabolic regulation on community diversity, ecosystem functions and evolutionary dynamics. By incorporating an ecological perspective into metabolic engineering strategies, researchers can optimise plant performance while ensuring ecosystem stability and biodiversity, thus fostering the coexistence of sustainable agriculture and natural systems.

### 4.4 Applications of Emerging Technologies

Emerging technologies, such as single‐cell metabolomics and advanced sensing systems, are revolutionising the study of plant–virus interactions by providing unprecedented tools to elucidate how viruses reprogramme plant metabolic networks and cellular dynamics. These high‐resolution and real‐time monitoring techniques enable researchers to uncover the metabolic adaptation processes in plants under viral stress, paving the way for the development of targeted antiviral strategies.

Traditional metabolomics studies, which rely on bulk tissue‐level analyses, often overlook cell‐to‐cell variations in metabolic responses during viral infection. Single‐cell metabolomics overcomes this limitation by capturing metabolic changes at the single‐cell level, providing a refined view of viral reprogramming in plant metabolic pathways (Kumar et al. 2020). For example, viral infections induce the localised accumulation of defence metabolites in specific cells, while others may exhibit suppressed metabolic activity. These cell‐specific differences highlight the complexity of virus–host interactions and provide new targets for antiviral defence.

Advancements in mass spectrometry imaging (MSI) have significantly improved the spatial visualisation of metabolite distributions in plant tissues. MSI enables the mapping of defence metabolite accumulation near viral infection sites, offering insights into localised immune responses (Zou et al. 2025; Lu et al. 2024). Additionally, this technique has revealed how plants spatially regulate secondary metabolite synthesis, such as phenylpropanoids and flavonoids, to counteract viral invasion and spread.

Non‐invasive sensing technologies have further enhanced our ability to monitor plant physiological states in real time. High‐sensitivity biosensors and spectral imaging technologies can detect subtle metabolic changes associated with viral infections, facilitating early diagnosis and targeted interventions (Zhang et al. 2025; Perdomo et al. 2024). For example, biosensors capable of monitoring SA dynamics have been developed to identify critical time points for activating immune responses. Similarly, non‐invasive MSI enables the continuous observation of metabolic fluxes under viral stress, providing insights into long‐term physiological adaptations (Sahin et al. 2020; Tan et al. 2024).

These emerging technologies provide ground‐breaking advancements for understanding plant–virus interactions at unprecedented resolutions and timescales. By integrating these tools with multi‐omics data, researchers can achieve dynamic and holistic insights into virus‐induced metabolic reprogramming. As single‐cell metabolomics and sensing systems continue to evolve, they will play a pivotal role in elucidating antiviral mechanisms, enabling precise metabolic engineering and facilitating the development of virus‐resistant crops to ensure global food security.

### 4.5 Integration of RNA Interference (RNAi) in Antiviral Strategies

The efficacy of RNAi‐based antiviral defence has been well demonstrated in various studies. One of the most notable applications is the use of transgenic approaches to confer resistance against plant viruses. For example, RNAi‐mediated resistance has been successfully developed in cassava plants against cassava brown streak virus, showcasing its practical potential in crop protection (Akbar et al. 2022).

However, despite its effectiveness, RNAi is not an absolute defence. Some viruses have evolved suppressors of RNA silencing to counteract this mechanism. A well‐documented example is the p19 protein in tombusviruses, which binds siRNAs and prevents them from guiding RISC to target viral RNAs. This arms race between plant defence mechanisms and viral counterdefences highlights the dynamic co‐evolution of host–pathogen interactions (Yang and Li 2018).

Beyond transgenic approaches, recent advancements have expanded the use of RNAi in plant protection. One promising alternative is the exogenous application of double‐stranded RNAs (dsRNAs), which trigger the RNAi pathway without the need for genetic modification. Studies have shown that synthetic dsRNAs applied directly to plants can effectively silence target genes in pathogens, providing a flexible and environmentally friendly alternative to conventional disease control methods (Halder et al. 2022).

Despite these advancements, several challenges remain for the widespread adoption of RNAi‐based antiviral strategies. Key issues include the stability and efficient delivery of dsRNAs in the field, potential off‐target effects and the emergence of RNAi‐resistant pathogen strains. Overcoming these obstacles will require interdisciplinary research and the integration of RNAi with other antiviral strategies, such as metabolic regulation and novel biotechnological approaches.

In conclusion, RNAi remains a powerful tool in combating plant viruses and other pathogens. By integrating RNAi with emerging technologies, researchers can develop durable and broad‐spectrum disease resistance strategies, contributing to the long‐term goal of global food security.

## 5 Conclusion

Plant viruses are pervasive worldwide, profoundly influencing agriculture settings and natural ecosystems by targeting a diverse range of hosts and manipulating their metabolic networks. These viral infections induce significant metabolic reprogramming in plants, particularly in carbohydrate, lipid, amino acid and secondary metabolic pathways, to facilitate replication and systemic spread, while simultaneously playing a pivotal role in plant defence, providing critical insights into the intricate interplay between plant metabolism and viral pathogenesis.

Advances in metabolomics and multi‐omics integration have shed light on these metabolic disruptions, revealing key regulatory pathways and potential biomarkers for viral resistance. Such findings have informed the development of modern antiviral strategies, including metabolic engineering and CRISPR/Cas9‐mediated gene editing, which hold great promise for breeding virus‐resistant crops. These innovations not only enhance plant resilience but also contribute to reducing dependency on chemical pesticides, promoting sustainable agricultural practices.

However, the ecological implications of metabolic regulation require careful consideration. Enhancing specific metabolic pathways to improve resistance may inadvertently affect ecosystem functions, biodiversity and plant interactions with beneficial organisms. Future research must address these challenges by incorporating ecological principles, systems biology and computational modelling to balance improved resistance with environmental sustainability.

Emerging technologies, such as single‐cell metabolomics, advanced sensing systems and machine learning‐driven multi‐omics integration, are set to revolutionise the understanding of virus–host interactions. These tools enable unprecedented resolution and dynamic monitoring of metabolic processes, offering new opportunities for precise metabolic engineering and targeted antiviral strategies.

## Conflicts of Interest

The authors declare no conflicts of interest.

## Data Availability

Data sharing is not applicable to this article as no new data were created or analysed.
